# The role of DC-SIGN as a trans-receptor in infection by MERS-CoV

**DOI:** 10.3389/fcimb.2023.1177270

**Published:** 2023-09-21

**Authors:** Nuria Labiod, Joanna Luczkowiak, María M. Tapia, Fátima Lasala, Rafael Delgado

**Affiliations:** ^1^ Department of Microbiology, Instituto de Investigación Hospital Universitario 12 de Octubre (Imas12), Madrid, Spain; ^2^ Departamento de Medicina, Facultad de Medicina, Universidad Complutense, Madrid, Spain; ^3^ CIBERINFEC, Instituto de Salud Carlos III, Madrid, Spain

**Keywords:** MERS-CoV, Coronaviruses, DC-SIGN, receptors, dendritic cell

## Abstract

DC-SIGN is a C-type lectin expressed in myeloid cells such as immature dendritic cells and macrophages. Through glycan recognition in viral envelope glycoproteins, DC-SIGN has been shown to act as a receptor for a number of viral agents such as HIV, Ebola virus, SARS-CoV, and SARS-CoV-2. Using a system of Vesicular Stomatitis Virus pseudotyped with MERS-CoV spike protein, here, we show that DC-SIGN is partially responsible for MERS-CoV infection of dendritic cells and that DC-SIGN efficiently mediates trans-infection of MERS-CoV from dendritic cells to susceptible cells, indicating a potential role of DC-SIGN in MERS-CoV dissemination and pathogenesis.

## Introduction

In the last 20 years, three coronaviruses have been identified as highly pathogenic for humans, which are severe acute respiratory syndrome coronavirus (SARS-CoV), Middle East respiratory syndrome coronavirus (MERS-CoV), and severe acute respiratory syndrome coronavirus 2 (SARS−CoV−2). In June 2012, a highly pathogenic coronavirus was identified at a hospital in Jeddah, Saudi Arabia, and later it was named Middle East respiratory syndrome coronavirus (MERS-CoV) (Zaki et al., 2012). The mortality rate of the virus was estimated at approximately 40% (Al Awaidy & Khamis, 2019). Dipeptidyl peptidase 4 (DPP4) has been demonstrated to be the functional cellular receptor for MERS-CoV (Raj et al., 2013). DPP4 or CD26 is primarily expressed on the surface of certain cell types, including epithelial cells in the respiratory tract, endothelial cells, and immune cells such as monocyte-derived DC (Mo-DC) (Scobey et al., 2013; Zhong et al., 2013; Chu et al., 2014). The importance of viral receptors has been thoroughly researched and it is a key factor in interpreting the pathogeny of the virus (Raj et al., 2013).

In these scenarios, the viral receptors present on dendritic cells (DCs) become interesting. DCs are found in the skin and mucosal tissue (van Kooyk, 2008) and circulate through extracellular spaces, actively surveying the presence of foreign antigens. They are considered the first line of defense against pathogenic invasion due to their abundant expression of various receptors on their cell surface. These include pattern recognition receptors (PRRs) such as Toll-like receptors (TLRs) and C-type lectin receptors (CLRs) that recognize structures with a high mannose content (Lundberg & Rydnert, 2014).

It has been evidenced that the expression of the C-type lectin DC-SIGN (Dendritic cell-specific ICAM-3-grabbing nonintegrin) on dendritic cells plays an important role in the entry and dissemination processes of various viruses, such as the human immunodeficiency virus (HIV), which is one of the most studied cases. Conventional HIV infection involves virus binding to the CD4 receptor, followed by interaction with co-receptors such as CCR5 or CXCR4 on CD4+ T lymphocytes to initiate direct infection or cis infection. However, there is an alternative pathway. In dendritic cells, DC-SIGN recognizes the high mannose region of the HIV gp120 protein, conferring upon dendritic cells the ability to facilitate trans-infection. This means that dendritic cells can capture the virus and transmit it to susceptible cells that express the necessary receptors for viral entry. It has been shown that this mechanism represents a significant route of virus dissemination from mucosal surfaces to replication in T cells present in secondary lymphoid organs through the interaction of DC-SIGN with immature dendritic cells (Geijtenbeek et al., 2000).

These host binding factors also play a crucial role in viral infection mechanisms as they increase the density of viral particles on cell surfaces, promoting their adhesion and consequently facilitating access to the real receptors (Thépaut et al., 2021). These mechanisms that facilitate trans-infection, where secondary presentation through non-permissive cells avoid immunological recognition, play a relevant role not only in HIV but have also been described in other viruses such as SARS-CoV, SARS-CoV-2, Ebola virus, or Dengue (Alvarez et al., 2002; Tassaneetrithep et al., 2003; Jeffers et al., 2004; Léger et al., 2016; Guo et al., 2021; Lempp et al., 2021; Thépaut et al., 2021).

Therefore, this study aimed to explore the role of DC-SIGN present in dendritic cells in the viral infection and dissemination mechanisms of MERS-CoV.

## Materials and methods

### Cell lines

African Green Monkey Cell Line (Vero E6) and Baby hamster kidney cells (BHK-21, 12-14-17 MAW, Kerafast, Boston, MA) were cultured in Dulbecco´s modified Eagle medium (DMEM) supplemented with 10% heat-inactivated fetal bovine serum (FBS), 25 μg/mL gentamycin, and 2 mM L-glutamine.

### Production of human monocyte-derived dendritic cells

Blood samples were obtained from healthy human donors (Hospital Universitario 12 de Octubre, Madrid, Spain) subject to informed consent and IRB approval. Peripheral blood mononuclear cells (PBMCs) were isolated by Ficoll density gradient centrifugation (Ficoll Paque, GE17-5442-02, Sigma-Aldrich). To generate monocyte-derived dendritic cells (MDDCs), CD14+ monocytes were purified using anti-human CD14 antibody-labeled magnetic beads and iron-based LS columns (Miltenyi Biotec) and used directly for further differentiation into dendritic cells (Domínguez-Soto et al., 2011). Differentiation to immature MDDCs was achieved by incubation at 37°C with 5% CO2 for 7 days and subsequent activation with cytokines GM-CSF (1000 U/mL) and IL-4 (500 U/mL) (Miltenyi Biotec) every other day.

### Production of pseudotyped recombinant Vesicular Stomatitis Virus

rVSV-luc pseudotypes (PSV) were generated following a published protocol (Whitt, 2010). First, BHK-21cells were transfected using Lipofectamine 3000 (Fisher Scientific) to express the different viral envelope glycoproteins: Ebola virus Makona Glycoprotein (EBOV-GP, GenBank KM233102.1) and S protein of MERS-CoV (NC_019843) were synthesized and cloned into pcDNA3.1 by GeneArt AG technology (Life Technologies, Regensburg, Germany); S protein of SARS-CoV-2 (codon-optimized; kindly provided by J. García-Arriaza, CNB-CSIC, Spain) or S protein of SARS-CoV (SinoBiological, Cat: VG40150-G-N) were cloned into a pCMV/hygro vector. After 24 h, transfected cells were inoculated with a replication-deficient rVSV-luc pseudotype (MOI: 3–5) (Kerafast, Boston, MA). After 1 h incubation at 37°C, cells were washed exhaustively with PBS and then DMEM supplemented with 5% heat-inactivated FBS, and 25 μg/mL gentamycin and 2 mM L-glutamine were added. PSV were collected 24 h post-inoculation, clarified from cellular debris by centrifugation, and stored at -80°C. Infectious titers were estimated as tissue culture infectious doses by limiting dilution of rVSV-luc-pseudotypes on Vero E6 cells. Luciferase activity was determined by luciferase assay (Steady-Glo Luciferase Assay System, Promega).

### Antibodies and inhibitors

The antibodies used in the experiments were Mouse IgG2a Isotype Control (Invitrogen), MERS Coronavirus Spike Protein neutralizing Antibody MA5-29975 (Invitrogen), Invitrogen CD26 Monoclonal Antibody (BA5b) (Invitrogen), Human DC-SIGN+DC-SIGNR Antibody (R&D Systems), PE anti-human CD209 (DC-SIGN) Antibody (Biolegend), and CD26 Monoclonal Antibody (BA5b) PE (Invitrogen). As a lectin-binding control, mannan from Saccharomyces cerevisiae (Sigma-Aldrich) was used. Mannan is used in experiments to inhibit C-type lectin receptors, such as DC-SIGN due to its ability to block the interaction between these receptors and viral or pathogenic ligands. Being a complex polysaccharide, mannan competes with the binding sites of the receptors, acting as a decoy and preventing the attachment of infectious agents to host cells (Jan et al., 2018).

### Direct (in cis) cellular infection with PSV

**Figure 1:**
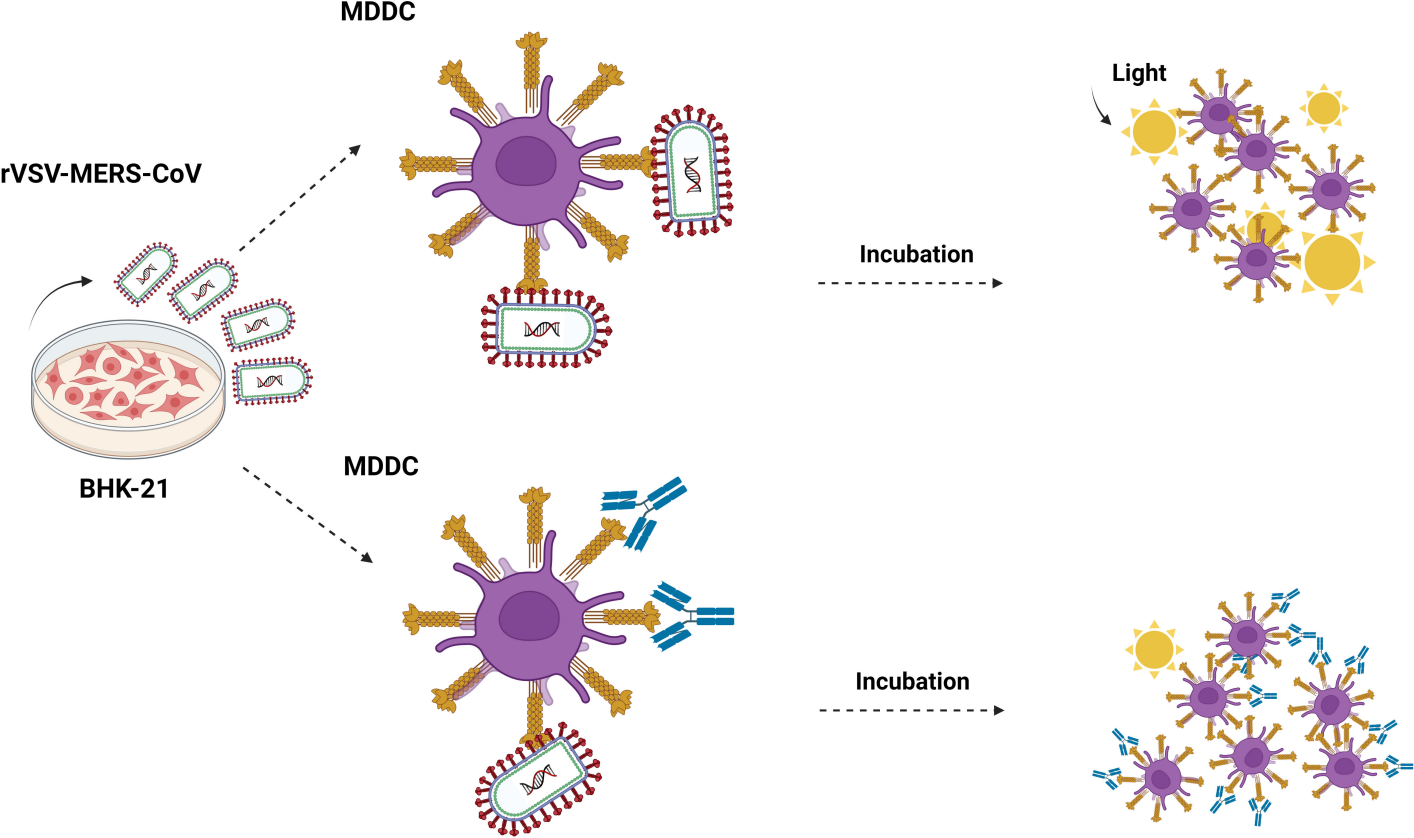
Cis-infection in monocyte-derived dendritic cells. Outline of the direct infection assay. MDDC cells are exposed to recombinant rVSV MERS-CoV, EBOV, SARS-CoV, and SARS-CoV-2 pseudotypes, and also in the presence of mannan, isotype control antibody, anti-DC/L-SIGN, anti-DPP4, and neutralizing anti-MERS antibodies. After 24 hours, the cells are washed and lysed for the luciferase assay. Made with Biorender.com.

primary cells MDDCs (5x10^4^) were challenged with MERS-CoV, SARS-CoV, SARS-CoV-2, EBOV-GP pseudotyped recombinant viruses (MOI: 0.5–2), mannan, and different antibodies (**Figure 1**). After 24 h of incubation, cells were washed twice with PBS and lysed for luciferase assay.

### Trans-infection assays with PSV

**Figure 2:**
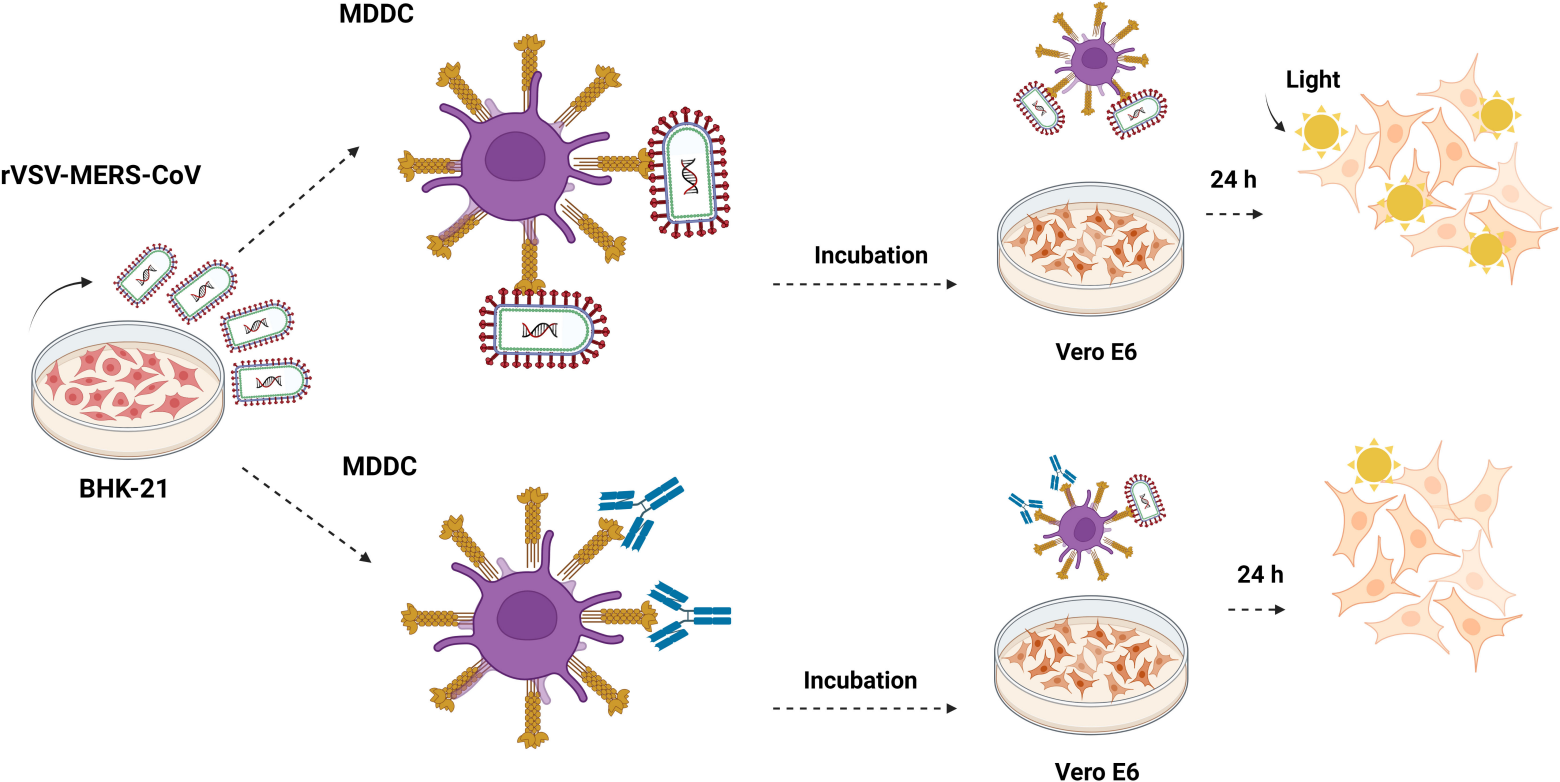
Trans-infection in monocyte-derived dendritic cells. Outline of the trans-infection assay. MDDC cells are exposed to recombinant rVSV MERS-CoV, EBOV, SARS-CoV, and SARS-CoV-2 pseudotypes, and also in the presence of mannan, isotype control antibody, anti-DC/L-SIGN, anti-DPP4, and neutralizing anti-MERS antibodies and incubated for 2h. After the incubation time, cells were centrifuged at 1200 rpm for 5 minutes and washed six times with PBS. MDDCs were then resuspended in RPMI medium and co-cultivated with adherent Vero E6 cells. After 24 hours, the cells were washed and lysed for the luciferase assay. They were made with Biorender.com.

For trans-infection studies, MDDCs (5x10^4^) were challenged with recombinant MERS-CoV, SARS-CoV, SARS-CoV-2, or EBOV-GP pseudotyped viruses (MOI: 0.5–2) and incubated for 2h with rotation (**Figure 2**). The antibodies were incubated with the MDDC cells 40 minutes prior to virus exposure, except for the neutralizing antibody against MERS, which was incubated with the virus for 40 minutes before the addition of the cells. After the incubation time, cells were then centrifuged at 1200 rpm for 5 minutes and washed six times with PBS supplemented with 0.5% bovine serum albumin (BSA) and 1 mM CaCl2 to ensure the removal of unbound virus from the cell receptor. The inclusion of CaCl2 in the wash buffer helps to maintain the integrity and functionality of DC-SIGN during the experimental procedures. After that, MDDCs were then resuspended in RPMI medium and co-cultivated with adherent Vero E6 cells (1.5x10^5^ cells/well) on a 24-well plate. After 24h, the supernatant was removed and the monolayer of Vero E6 was washed with PBS six times and lysed for luciferase assay. These washes were performed to remove the co-cultivated dendritic cells from the Vero E6 cells and ensure that the infection observed in the Vero E6 cells was due to the trans-infection process and not direct infection that may occur in the MDDC cells. In order to control this variable, a parallel trans-infection experiment with MERS-CoV was also conducted on MDDC cells under the same conditions. After the incubation period, the dendritic cells were not co-cultivated with the Vero E6 target cells following the washes with PBS. The levels of RLU/s in the infection readout after 24 hours indicate the amount of MERS-CoV that had successfully entered through direct infection in the dendritic cells during the two-hour incubation period.

### Verification of DC-SIGN’s role as a trans-receptor for MERS-CoV

The duration of migration for a dendritic cell from the mucosal layers where MERS-CoV could potentially adhere to a deeper tissue where infection initiation occurs remains uncertain. To enhance our comprehension of this biological phenomenon, a trans-infection assay was conducted, incorporating varying time intervals between attachment and infection. In order to monitor the viral entry of MERS-CoV into MDDCs during these incubation periods, an infection assay was carried out under identical conditions. Trypsin treatment: To discern between viral attachment and entry into the cell, a trypsin treatment experiment (Hanley et al., 2010) was conducted on MDDCs both before and after exposure to the MERS-CoV virus. This was performed to ascertain whether trans-infection was stopped.

## Results

### DC-SIGN does not act as a direct receptor for MERS-CoV

MERS-CoV PSV demonstrated a clear capability to infect MDDC in cis, and levels of infection were reduced in the presence of anti-DC/L-SIGN although it was not significant, and it was reduced by mannan (**Figure 1**). Cis infection of MDDC with SARS-CoV-1 and SARS-CoV-2 PSV displayed a total absence of infection, whereas EBOV PSV exhibited clear infection of MDDC that was inhibited by anti-DC-SIGN as demonstrated before (Alvarez et al., 2002).

**Figure 3:**
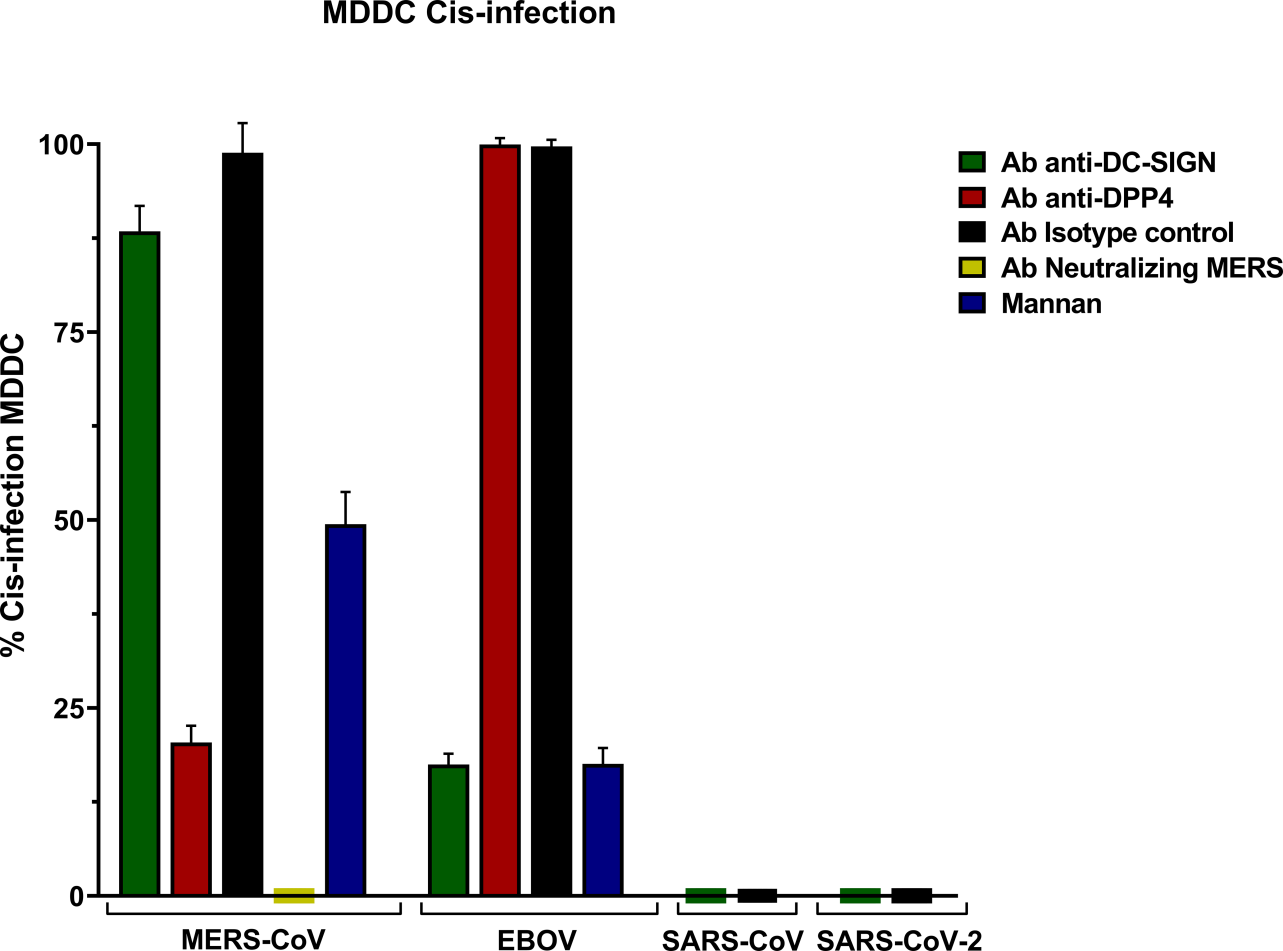
Cis-infection in Monocyte-derived Dendritic Cells (MDDC) with pseudoviruses based in rVSV-luc. Percentage of cis-infection in MDDCs, with SARS-CoV-2, SARS-CoV, MERS-CoV, and EBOV-GP, compared with infection in the absence of antibodies. Bars represent the mean SEM of the mean of four independent experiments with cells from two different donors performed in duplicates. Data were compared among multiple independent groups using one-way ANOVA (P= <0,0001) using GraphPad Prism v8. Cis-infection values are expressed as Percentage (%).

The isotype control antibody employed alongside the anti-DC/L-SIGN and anti-DPP4 antibodies confirmed the specificity of the primary antibody binding, ruling out any potential effects from other protein interactions or nonspecific Fc receptor binding. Cis infection by MERS-CoV in the presence of anti-DPP4 antibody exhibited a significant reduction, thereby demonstrating the utilization as a direct cellular receptor by MERS-CoV. Infection with MERS-CoV was completely abolished in the presence of its neutralizing antibody (**Figure 3**).

The results showed percentages (%) considering cis-infection in the absence of antibodies as 100% of infection.

To evaluate the effectiveness of these antibodies, a cis infection assay was conducted in human 293T cells and African green monkey Vero E6 cells.

The results showed that 293T cells were infected by MERS-CoV and SARS-CoV-2. MERS-CoV and SARS-CoV-2 infections in 293T were not inhibited by isotype control antibody; however, infection with MERS-CoV in the presence of the anti-DPP4 antibody showed a significant reduction, unlike SARS-CoV-2 where the infection persisted. Additionally, MERS-CoV infection has been inhibited in the presence of the neutralizing antibody (**Supplementary Figure 1**).

On the other hand, cis-infection showed that Vero E6 cells were infected by MERS-CoV and SARS-CoV-2 and this infection was not inhibited by isotype control antibody. Infection with MERS-CoV in the presence of the anti-DPP4 antibody did not show a significant reduction due to the anti-DPP4 antibody being a human antibody. Additionally, MERS-CoV infection has been inhibited in the presence of the neutralizing antibody (**Supplementary Figure 2**).

### DC-SIGN acts as a trans-receptor for MERS-CoV

**Figure 4:**
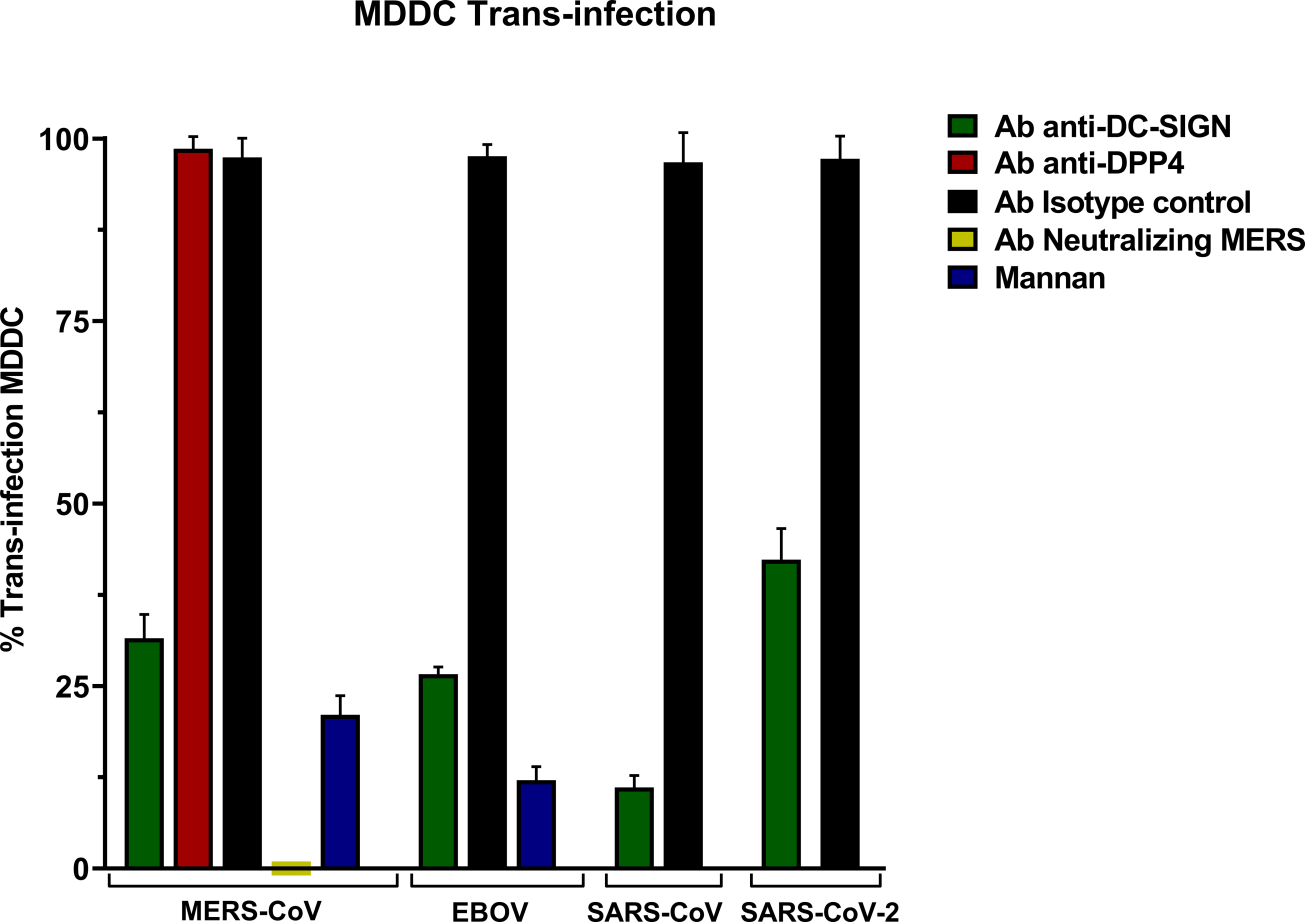
Trans-Infection in Monocyte-derived Dendritic Cells (MDDC) with pseudoviruses based in rVSV-luc. Percentage of trans-infection in MDDCs with SARS-CoV-2, SARS-CoV, MERS-CoV, and EBOV-GP compared with trans-infection in the absence of antibodies. Bars represent the mean SEM of the mean of four independent experiments with cells from two different donors performed in duplicates. Data were compared among multiple independent groups using one-way ANOVA (P= <0,0001) using GraphPad Prism v8. Trans-infection values are expressed as Percentage (%).

The trans-infection assay in MDDC with MERS-CoV PSV led to high levels of infection and anti-DC-SIGN exhibited a significant reduction in infectivity showing that DC-SIGN promoted an efficient trans-infection by MERS-CoV from MDDC to Vero E6 cells (**Figure 4**). Similar DC-SIGN-specific trans-infection was also shown for SARS-CoV, SARS-CoV-2, and EBOV PSV.

An isotype control antibody was used for both anti-DC/L-SIGN and anti-DPP4 antibodies to confirm that the binding of the primary antibody is specific and not the result of other protein interactions or non-specific binding of the Fc receptor. The trans-infection mediated by MERS-CoV was entirely suppressed when its neutralizing antibody was present, and the control of inhibition of infection mediated by lectin receptors mannan reduced the trans-infection. Finally, trans-infection by MERS-CoV in the presence of the anti-DPP4 antibody did not show a significant reduction.

On a separate note, to control the viral entry of MERS-CoV into MDDCs during these incubation periods, a concurrent trans-infection experiment involving MDDC cells was also carried out using identical conditions. Subsequent to the incubation phase, the dendritic cells were not co-cultured with the Vero E6 target cells after PBS washes. The levels of relative light units per second (RLU/s) in the infection readout after 24 hours demonstrated minimal infection by MERS-CoV, in comparison with the levels of trans-infection in Vero E6 (**Supplementary Figure 3**).

To further explore the role of DC-SIGN as a trans-receptor for MERS-CoV, an infection assay was conducted under identical conditions at different time points. The assay demonstrated effective trans-infection at 2, 6, and 12 hours, with the highest binding observed at the six-hour mark. The parallel assay in MDDCs revealed that viral entry into the cells increases with the incubation time, albeit remaining modest in comparison to the levels of trans-infection observed in Vero E6 cells (**Supplementary Figure 4**).

The results of the trypsin treatment experiment followed by PBS washing demonstrated that trans-infection is halted when MDDCs are treated both before exposure to the MERS-CoV virus and after virus attachment. There were no significant differences between pre-incubation and post-incubation trypsin treatment during the trans-infection assay (**Supplementary Figure 5**).

### Analysis of receptor expression levels

PE anti-human CD209 (DC-SIGN) Antibody (Biolegend) and CD26 Monoclonal Antibody (BA5b) PE (Invitrogen) were employed to quantify the expression of the DC-SIGN receptor (**Supplementary Figure 6**) and DPP4 receptor (**Supplementary Figure 7**) on Monocyte-Derived Dendritic Cells (MDDCs). The expression was analyzed by flow cytometry in a Cytek Aurora with FlowJo V10.7.1 software.

## Discussion

We studied the interaction of DC-SIGN with MERS-CoV. DC-SIGN, also named CD209 or dendritic cell-specific (DC) intercellular adhesion molecule-3-grabbing non-integrin (ICAM-3), is expressed in immature dendritic cells of peripheral tissues that we can find in diverse places such as the tonsils, mucous tissues, lymph nodes, monocyte-derived DCs, and spleen (Geijtenbeek et al., 2002). This type-C lectin recognizes highly glycosylated ligands present in pathogens such as viruses and human tissues through the recognition of carbohydrates mediated by the receptor’s CDR (Soilleux et al., 2002). It has been proved that DC-SIGN expressed in DCs has an important role in the entry and dissemination of different viruses like the known case of the human immunodeficiency virus (HIV). Even though DC-SIGN is not a direct receptor of this virus for the viral entry for it does not mediate in the host cell, it confers to DCs the ability to facilitate trans-infection, that is, to capture the virus and transmit it to permissive cells that express the necessary receptors for the viral entry. This mechanism has been demonstrated as an important route for the dissemination of the virus from mucous surfaces to its replication in T cells present in secondary lymphoid organs by binding to DC-SIGN in immature DCs (Geijtenbeek et al., 2000). These binding factors of the host are key elements in viral infection mechanisms for they increase the density of viral particles at the cell surfaces, increasing its adhesion to them and thus boosting the access to their real receptors (Thépaut et al., 2021). These mechanisms facilitate trans-infection where a secondary presentation by non-permissive cells evading the immunological recognition system plays a role not only for HIV but for other viruses as well.

Even though in the bibliography the interaction between DC-SIGN lectin and MERS-CoV is described (Campana et al., 2020; Rahimi, 2020; Chapoval & Keegan, 2021), until now no study has experimentally proved such interaction. We have studied if MERS-CoV, whose canonical receptor is dipeptidyl peptidase-4 protein (DPP4), could have DC-SIGN as an alternative receptor for cis or trans-infection.

A study by Chu et al. (2014) proved productive infection by MERS in dendritic cells. Those patients who were infected with MERS-CoV, in clinical terms, developed coagulopathy, multiorgan dysfunction, lymphopenia, neutrophilia, and thrombocytopenia. It is worth mentioning that the virus is not only detected in the respiratory system but also in the urine, blood, and feces of infected people, which would support a systemic dissemination of the virus. For this reason, in people infected with MERS-CoV, monocyte-derived macrophages and dendritic cells could act as potential dissemination carriers by facilitating viral spread to the lymph nodes where, due to the interaction with T cells, they would boost the storm of cytokines and/or chemokines, imitating the scenery in SARS-CoV-1, worsening the clinical profile of the infected individual. In that study, they proved the existence of a productive infection in dendritic cells by MERS-CoV, which is something that does not happen in SARS-CoV (Chu et al., 2014).

The present study aims to explore if MERS-CoV can use DC-SIGN as a direct receptor, in this way partially explaining the productive infection demonstrated in dendritic cells in the direct infection of the virus discussed by Chu et al., or if it can also act, as it does in SARS-CoV-1 and SARS-CoV-2, as a binding factor, as well as its possible role in viral dissemination.

Cis-infection showed infection of dendritic cells by MERS-CoV PSV; however, anti-DC-SIGN could not significantly reduce infection indicating that this is not dependent on DC-SIGN though the decrease in the levels of infection in the presence of mannan indicates that a lectin receptor might be involved.

Trans-infection assays with MDDCs showed an increase in the infection values of target Vero E6 cells and a significant decrease in the presence of anti-DC-SIGN, indicating that the lectin is acting as a trans-receptor for MERS-CoV. The exact mechanism of how trans-infection occurs and the subsequent spread of the virus to other cells is still being studied. It is possible that the virus, once captured by dendritic cells, can be transported to other cells through various mechanisms, such as cell-to-cell contact or the release of viral particles.

The biological relevance of the interaction of DC-SIGN with MERS-CoV through the trans-infection process in the pathogenesis of severe respiratory disease remains to be explored.

## Data availability statement

The raw data supporting the conclusions of this article will be made available by the authors, without undue reservation.

## Ethics statement

The studies involving humans were approved by Comité Investigación Clínica Hospital Universitario 12 de Octubre (Reference CEIm 20/157). The studies were conducted in accordance with the local legislation and institutional requirements. The human samples used in this study were acquired from Blood samples were obtained from healthy human donors (Hospital Universitario 12 de Octubre, Madrid, Spain) under informed consent and IRB approval. Written informed consent for participation was not required from the participants or the participants’ legal guardians/next of kin in accordance with the national legislation and institutional requirements.

## Author contributions

NL performed the experiments. JL, FL and MMT collaborated in research. RD directed the investigation. NL and RD wrote the paper. All authors contributed to the article and approved the submitted version.
