# Supplementary material for: The role of DC-SIGN as a trans-receptor in infection by MERS-CoV
Source: Front Cell Infect Microbiol. 2023 Sep 21;13:1177270. doi: 10.3389/fcimb.2023.1177270 (PMC10552186; doi:10.3389/fcimb.2023.1177270)
Supplement: Supplementary file 1 [file DataSheet_1.pdf]

Supplementary Material

Supplementary Figures

S1

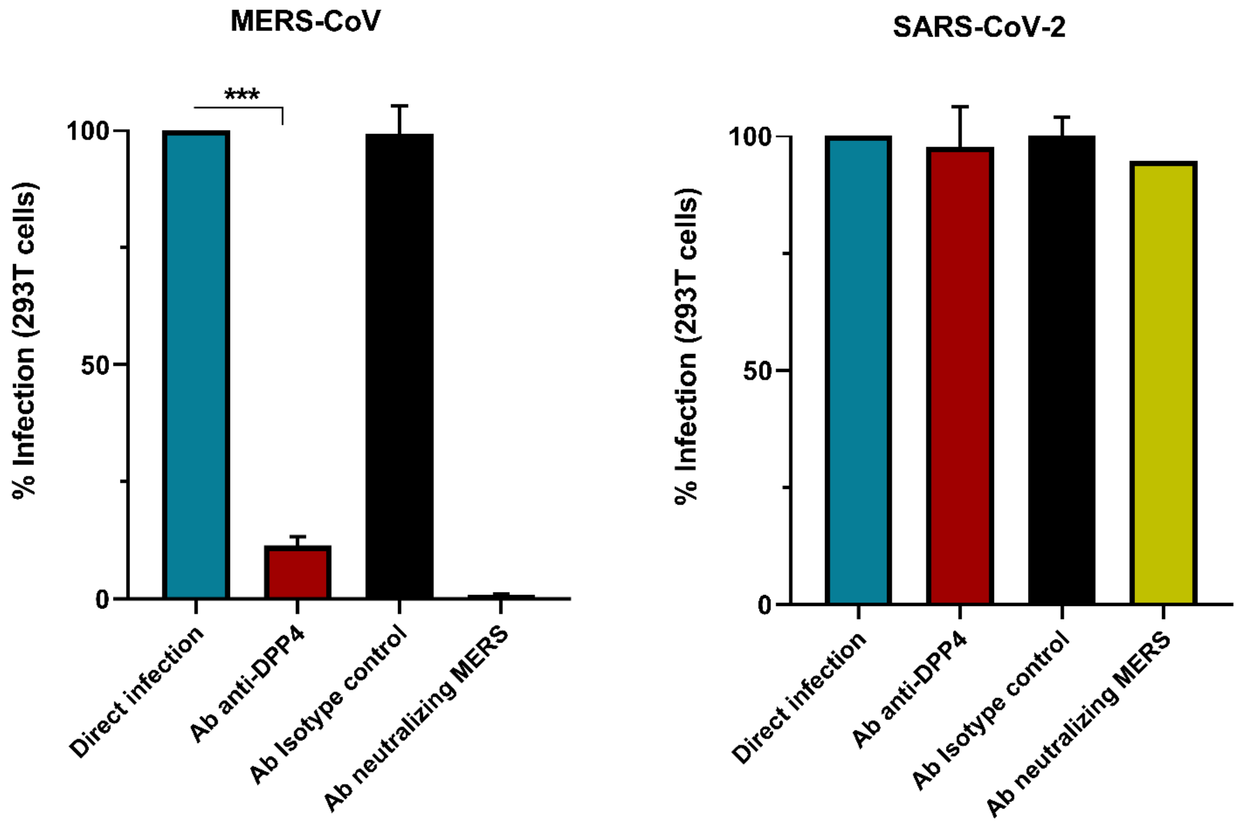

Figure S1. Cis-infection in 293T cells with pseudoviruses based in rVSV-luc. Percentage of direct infection with MERS-CoV and SARS-CoV-2 pseudoviruses as compared with direct infection in the absence of antibodies. Bars represent mean SEM of mean of duplicates. Infection values are expressed as Percentage (%). Statistical significance was calculated by t test using GraphPad Prism v8.

S2

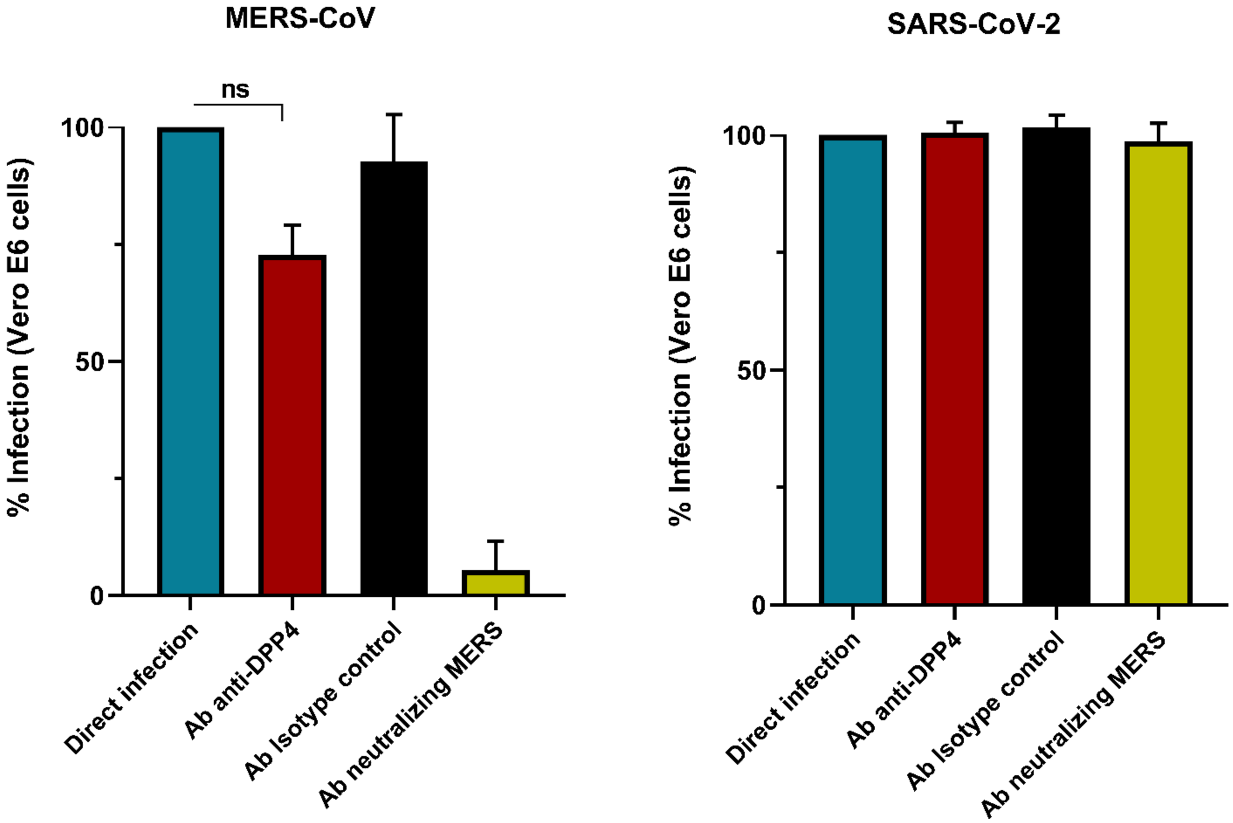

Figure S2. Cis-infection in Vero E6 cells with pseudoviruses based in rVSV-luc. Percentage of direct infection with MERS-CoV and SARS-CoV-2 pseudoviruses as compared with direct infection in the absence of antibodies. Bars represent mean SEM of mean of duplicates. Infection values are expressed as Percentage (%). Statistical significance was calculated by t test using GraphPad Prism v8.

S3

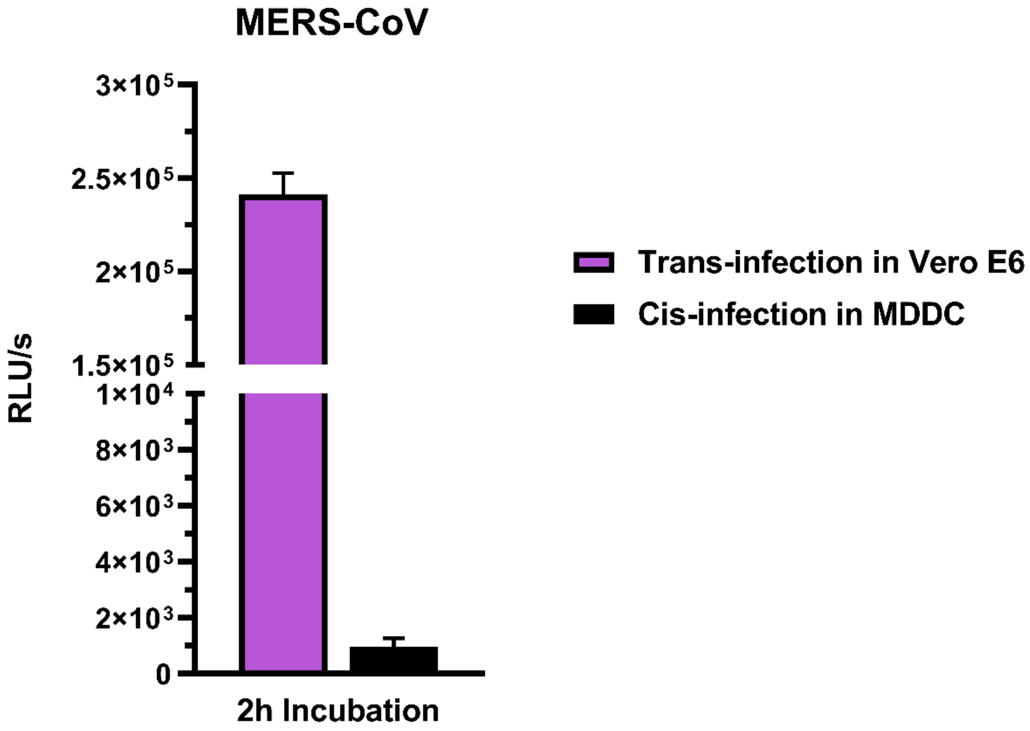

Figure S3. Verification of DC-SIGN's role as a transreceptor for MERS-CoV. The role of DC-SIGN as a transreceptor for MERS-CoV was confirmed in Vero E6 cells (purple bar) through a parallel viral entry assay in Monocyte-Derived Dendritic Cells (MDDCs) over a two-hour trans-infection period following PBS washes (black bar). Bars represent mean SEM of mean of 2 independent experiments with cells from 2 different donors performed in duplicates. Infection values are expressed as Relative Light Units (RLUs).

S4

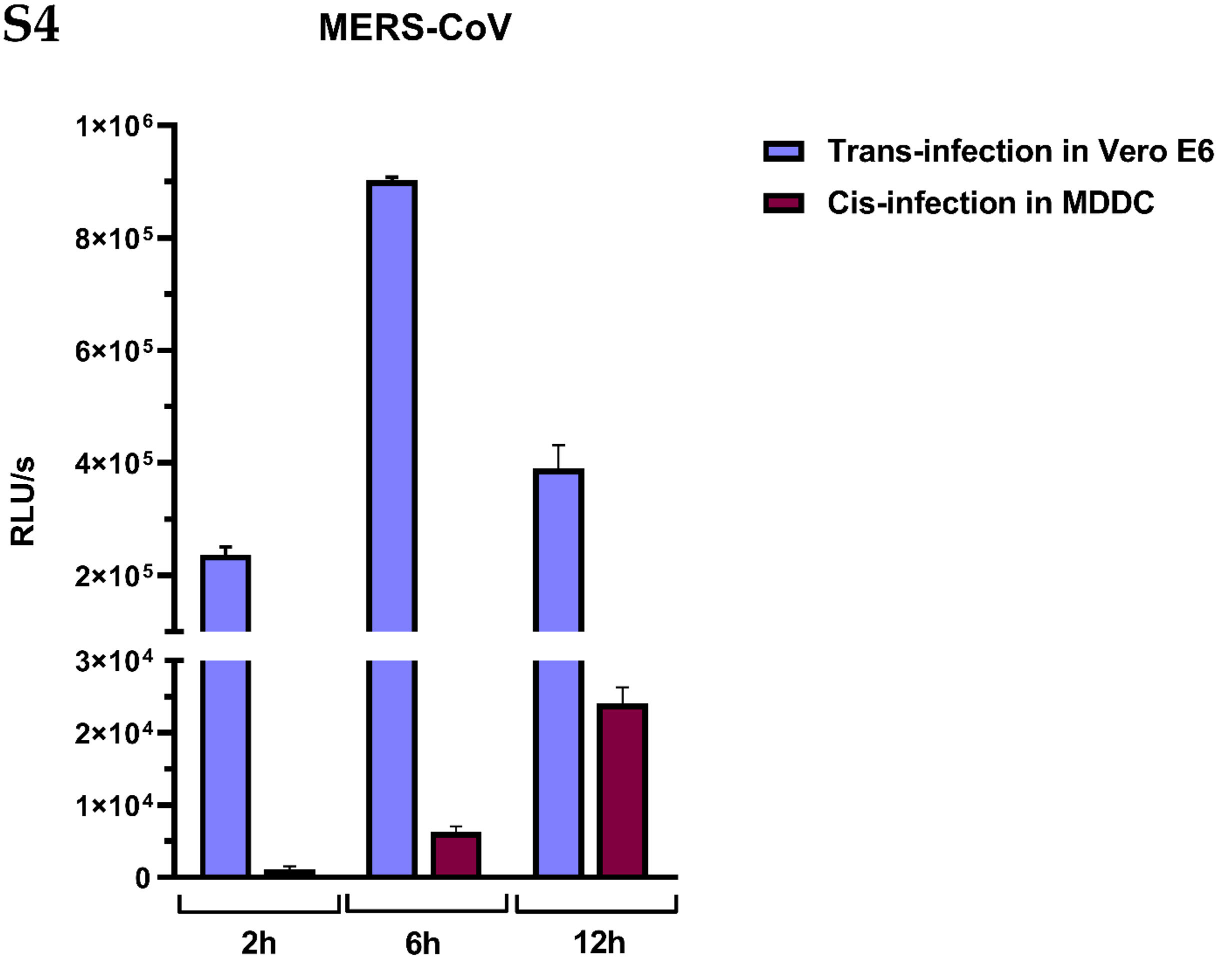

Figure S4. Trans-infection assay with an extended time course between attachment and infection. Trans-infection assay in Monocyte-Derived Dendritic Cells (MDDCs) was conducted at 2, 6 and 12 hours of incubation with MERS-CoV (lilac bar) before contact with Vero E6 cells. In parallel, a cis infection assay was performed to monitor the viral entry of MERS-CoV into MDDCs during attachment at the various incubation times (red bar). Bars represent standard errors of the mean (SEM). Infection values are expressed as Relative Light Units (RLUs).

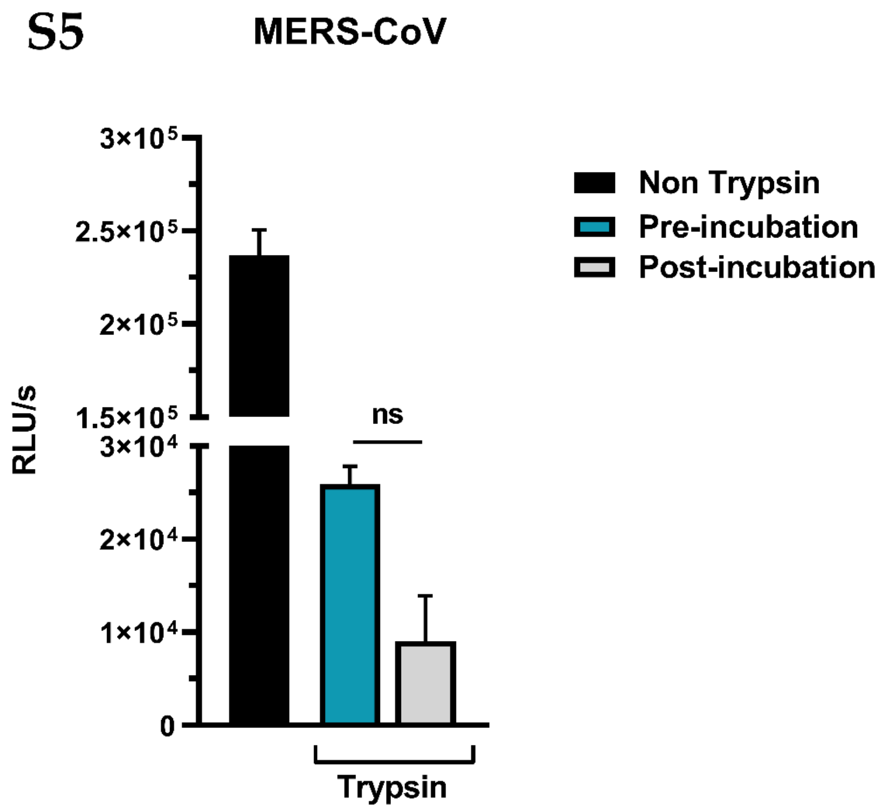

Figure S5. Trypsin treatment assay in trans-infection of Monocyte-Derived Dendritic Cells (MDDCs) in Vero E6 cells. Bars represent standard errors of the mean (SEM). Infection values are expressed as Relative Light Units (RLUs). Statistical significance was calculated by Wilcoxon test using GraphPad Prism v8.

S6

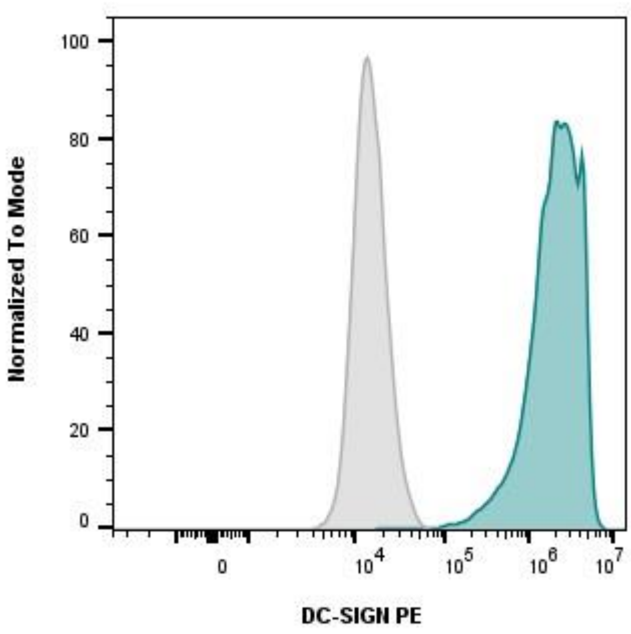

Figure S6. DC-SIGN expression in Monocyte-derived Dendritic Cells (MDDC) by flow cytometry analysis. The colored cell population represents MDDCs stained with Anti-DC-SIGN_PE. The expression of DC-SIGN in MDDCs is >95%. In grey unstained MDDCs. DC-SIGN expression was analyzed by flow cytometry in a Cytek Aurora with FlowJo V10.7.1 software.

S7

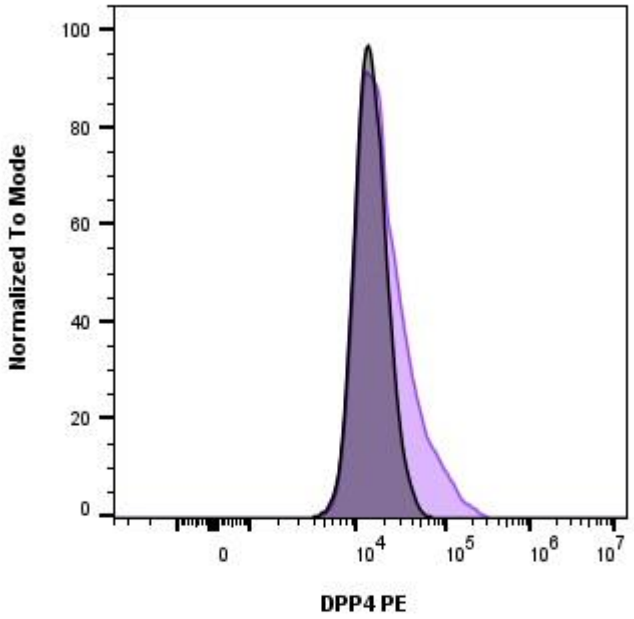

Figure S7. DPP4 expression in Monocyte-derived Dendritic Cells (MDDC) by flow cytometry analysis. The colored cell population represents MDDCs stained with Anti-DPP4/CD26_PE. The expression of DPP4 in MDDCs is 14%. In grey unstained MDDCs. DPP4 expression was analyzed by flow cytometry in a Cytek Aurora with FlowJo V10.7.1 software.
